# Supplementary material for: A Critical View of the Application of the APEX Software (Aqueous Photochemistry of Environmentally-Occurring Xenobiotics) to Predict Photoreaction Kinetics in Surface Freshwaters
Source: Molecules. 2019 Dec 18;25(1):9. doi: 10.3390/molecules25010009 (PMC7017383; doi:10.3390/molecules25010009)
Supplement: Supplementary file 1 [file molecules-25-00009-s001.zip › 3.2.4_gcc-4.4.0.zip!/3.2.4_gcc-4.4.0/3.2.4_gcc-4.4.0/doc/octave/html/interpreter/Controlling-Subprocesses.html]

Controlling Subprocesses - Untitled


Next: Process ID Information,
Previous: Networking Utilities,
Up: System Utilities


---

### 34.5 Controlling Subprocesses

Octave includes some high-level commands like `system` and
`popen` for starting subprocesses. If you want to run another
program to perform some task and then look at its output, you will
probably want to use these functions.

Octave also provides several very low-level Unix-like functions which
can also be used for starting subprocesses, but you should probably only
use them if you can't find any way to do what you need with the
higher-level functions.

— Built-in Function: **system** (string, return\_output, type)  
> Execute a shell command specified by string. The second
> argument is optional. If type is `"async"`, the process
> is started in the background and the process id of the child process
> is returned immediately. Otherwise, the process is started, and
> Octave waits until it exits. If the type argument is omitted, a
> value of `"sync"` is assumed.
>
> If two input arguments are given (the actual value of
> return\_output is irrelevant) and the subprocess is started
> synchronously, or if system is called with one input argument and
> one or more output arguments, the output from the command is returned.
> Otherwise, if the subprocess is executed synchronously, its output is
> sent to the standard output. To send the output of a command executed
> with system through the pager, use a command like
>
> ```
>           disp (system (cmd, 1));
> ```
>
> or
>
> ```
>           printf ("%s\n", system (cmd, 1));
> ```
>
> The `system` function can return two values. The first is the
> exit status of the command and the second is any output from the
> command that was written to the standard output stream. For example,
>
> ```
>           [status, output] = system ("echo foo; exit 2");
> ```
>
> will set the variable `output` to the string foo, and the
> variable `status` to the integer 2.

— Function File: [status, text] **unix** (command)  
— Function File: [status, text] **unix** (command, "-echo")  
> Execute a system command if running under a Unix-like operating
> system, otherwise do nothing. Return the exit status of the program
> in status and any output sent to the standard output in
> text. If the optional second argument `"-echo"` is given,
> then also send the output from the command to the standard output.
>
>
>
> **See also:** isunix, ispc, system.

— Function File: [status, text] = **dos** (command)  
— Function File: [status, text] = **dos** (command, "-echo")  
> Execute a system command if running under a Windows-like operating
> system, otherwise do nothing. Return the exit status of the program
> in status and any output sent to the standard output in
> text. If the optional second argument `"-echo"` is given,
> then also send the output from the command to the standard output.
>
>
>
> **See also:** unix, isunix, ispc, system.

— Function File: [output, status] = **perl** (scriptfile)  
— Function File: [output, status] = **perl** (scriptfile, argument1, argument2, ...)  
> Invoke perl script scriptfile with possibly a list of
> command line arguments.
> Returns output in output and status
> in status.
>
>
>
> **See also:** system.

— Built-in Function: fid = **popen** (command, mode)  
> Start a process and create a pipe. The name of the command to run is
> given by command. The file identifier corresponding to the input
> or output stream of the process is returned in fid. The argument
> mode may be
>
> `"r"`: The pipe will be connected to the standard output of the process, and open for reading. `"w"`: The pipe will be connected to the standard input of the process, and open for writing.
>
> For example,
>
> ```
>           fid = popen ("ls -ltr / | tail -3", "r");
>           while (ischar (s = fgets (fid)))
>             fputs (stdout, s);
>           endwhile
>                -| drwxr-xr-x  33 root  root  3072 Feb 15 13:28 etc
>                -| drwxr-xr-x   3 root  root  1024 Feb 15 13:28 lib
>                -| drwxrwxrwt  15 root  root  2048 Feb 17 14:53 tmp
> ```

— Built-in Function: **pclose** (fid)  
> Close a file identifier that was opened by `popen`. You may also
> use `fclose` for the same purpose.

— Built-in Function: [in, out, pid] = **popen2** (command, args)  
> Start a subprocess with two-way communication. The name of the process
> is given by command, and args is an array of strings
> containing options for the command. The file identifiers for the input
> and output streams of the subprocess are returned in in and
> out. If execution of the command is successful, pid
> contains the process ID of the subprocess. Otherwise, pid is
> −1.
>
> For example,
>
> ```
>           [in, out, pid] = popen2 ("sort", "-r");
>           fputs (in, "these\nare\nsome\nstrings\n");
>           fclose (in);
>           EAGAIN = errno ("EAGAIN");
>           done = false;
>           do
>             s = fgets (out);
>             if (ischar (s))
>               fputs (stdout, s);
>             elseif (errno () == EAGAIN)
>               sleep (0.1);
>               fclear (out);
>             else
>               done = true;
>             endif
>           until (done)
>           fclose (out);
>           waitpid (pid);
>                -| these
>                -| strings
>                -| some
>                -| are
> ```
>
> Note that `popen2`, unlike `popen`, will not "reap" the
> child process. If you don't use `waitpid` to check the child's
> exit status, it will linger until Octave exits.

— Built-in Function: val = **EXEC\_PATH** ()  
— Built-in Function: old\_val = **EXEC\_PATH** (new\_val)  
> Query or set the internal variable that specifies a colon separated
> list of directories to search when executing external programs.
> Its initial value is taken from the environment variable
> `OCTAVE_EXEC_PATH` (if it exists) or `PATH`, but that
> value can be overridden by the command line argument
> `--exec-path PATH`. At startup, an additional set of
> directories (including the shell PATH) is appended to the path
> specified in the environment or on the command line. If you use
> the `EXEC_PATH` function to modify the path, you should take
> care to preserve these additional directories.

In most cases, the following functions simply decode their arguments and
make the corresponding Unix system calls. For a complete example of how
they can be used, look at the definition of the function `popen2`.

— Built-in Function: [pid, msg] = **fork** ()  
> Create a copy of the current process.
>
> Fork can return one of the following values:
>
> > 0: You are in the parent process. The value returned from `fork` is the process id of the child process. You should probably arrange to wait for any child processes to exit. 0: You are in the child process. You can call `exec` to start another process. If that fails, you should probably call `exit`. < 0: The call to `fork` failed for some reason. You must take evasive action. A system dependent error message will be waiting in msg.

— Built-in Function: [err, msg] = **exec** (file, args)  
> Replace current process with a new process. Calling `exec` without
> first calling `fork` will terminate your current Octave process and
> replace it with the program named by file. For example,
>
> ```
>           exec ("ls" "-l")
> ```
>
> will run `ls` and return you to your shell prompt.
>
> If successful, `exec` does not return. If `exec` does return,
> err will be nonzero, and msg will contain a system-dependent
> error message.

— Built-in Function: [read\_fd, write\_fd, err, msg] = **pipe** ()  
> Create a pipe and return the reading and writing ends of the pipe
> into read\_fd and write\_fd respectively.
>
> If successful, err is 0 and msg is an empty string.
> Otherwise, err is nonzero and msg contains a
> system-dependent error message.

— Built-in Function: [fid, msg] = **dup2** (old, new)  
> Duplicate a file descriptor.
>
> If successful, fid is greater than zero and contains the new file
> ID. Otherwise, fid is negative and msg contains a
> system-dependent error message.

— Built-in Function: [pid, status, msg] = **waitpid** (pid, options)  
> Wait for process pid to terminate. The pid argument can be:
>
> −1: Wait for any child process. 0: Wait for any child process whose process group ID is equal to that of the Octave interpreter process. > 0: Wait for termination of the child process with ID pid.
>
> The options argument can be a bitwise OR of zero or more of
> the following constants:
>
> `0`: Wait until signal is received or a child process exits (this is the default if the options argument is missing). `WNOHANG`: Do not hang if status is not immediately available. `WUNTRACED`: Report the status of any child processes that are stopped, and whose status has not yet been reported since they stopped. `WCONTINUE`: Return if a stopped child has been resumed by delivery of `SIGCONT`. This value may not be meaningful on all systems.
>
> If the returned value of pid is greater than 0, it is the process
> ID of the child process that exited. If an error occurs, pid will
> be less than zero and msg will contain a system-dependent error
> message. The value of status contains additional system-dependent
> information about the subprocess that exited.
>
>
>
> **See also:** WCONTINUE, WCOREDUMP, WEXITSTATUS, WIFCONTINUED, WIFSIGNALED, WIFSTOPPED, WNOHANG, WSTOPSIG, WTERMSIG, WUNTRACED.

— Built-in Function: WCONINTUE **(**)  
> Return the numerical value of the option argument that may be
> passed to `waitpid` to indicate that it should also return
> if a stopped child has been resumed by delivery of a `SIGCONT`
> signal.
>
>
>
> **See also:** waitpid, WNOHANG, WUNTRACED.

— Built-in Function: **WCOREDUMP** (status)  
> Given status from a call to `waitpid`, return true if the
> child produced a core dump. This function should only be employed if
> `WIFSIGNALED` returned true. The macro used to implement this
> function is not specified in POSIX.1-2001 and is not available on some
> Unix implementations (e.g., AIX, SunOS).
>
>
>
> **See also:** waitpid, WIFEXITED, WEXITSTATUS, WIFSIGNALED, WTERMSIG, WIFSTOPPED, WSTOPSIG, WIFCONTINUED.

— Built-in Function: **WEXITSTATUS** (status)  
> Given status from a call to `waitpid`, return the exit
> status of the child. This function should only be employed if
> `WIFEXITED` returned true.
>
>
>
> **See also:** waitpid, WIFEXITED, WIFSIGNALED, WTERMSIG, WCOREDUMP, WIFSTOPPED, WSTOPSIG, WIFCONTINUED.

— Built-in Function: **WIFCONTINUED** (status)  
> Given status from a call to `waitpid`, return true if the
> child process was resumed by delivery of `SIGCONT`.
>
>
>
> **See also:** waitpid, WIFEXITED, WEXITSTATUS, WIFSIGNALED, WTERMSIG, WCOREDUMP, WIFSTOPPED, WSTOPSIG.

— Built-in Function: **WIFSIGNALED** (status)  
> Given status from a call to `waitpid`, return true if the
> child process was terminated by a signal.
>
>
>
> **See also:** waitpid, WIFEXITED, WEXITSTATUS, WTERMSIG, WCOREDUMP, WIFSTOPPED, WSTOPSIG, WIFCONTINUED.

— Built-in Function: **WIFSTOPPED** (status)  
> Given status from a call to `waitpid`, return true if the
> child process was stopped by delivery of a signal; this is only
> possible if the call was done using `WUNTRACED` or when the child
> is being traced (see ptrace(2)).
>
>
>
> **See also:** waitpid, WIFEXITED, WEXITSTATUS, WIFSIGNALED, WTERMSIG, WCOREDUMP, WSTOPSIG, WIFCONTINUED.

— Built-in Function: **WIFEXITED** (status)  
> Given status from a call to `waitpid`, return true if the
> child terminated normally.
>
>
>
> **See also:** waitpid, WEXITSTATUS, WIFSIGNALED, WTERMSIG, WCOREDUMP, WIFSTOPPED, WSTOPSIG, WIFCONTINUED.

— Built-in Function: **WNOHANG** ()  
> Return the numerical value of the option argument that may be
> passed to `waitpid` to indicate that it should return its
> status immediately instead of waiting for a process to exit.
>
>
>
> **See also:** waitpid, WUNTRACED, WCONTINUE.

— Built-in Function: **WSTOPSIG** (status)  
> Given status from a call to `waitpid`, return the number of
> the signal which caused the child to stop. This function should only
> be employed if `WIFSTOPPED` returned true.
>
>
>
> **See also:** waitpid, WIFEXITED, WEXITSTATUS, WIFSIGNALED, WTERMSIG, WCOREDUMP, WIFSTOPPED, WIFCONTINUED.

— Built-in Function: **WTERMSIG** (status)  
> Given status from a call to `waitpid`, return the number of
> the signal that caused the child process to terminate. This function
> should only be employed if `WIFSIGNALED` returned true.
>
>
>
> **See also:** waitpid, WIFEXITED, WEXITSTATUS, WIFSIGNALED, WCOREDUMP, WIFSTOPPED, WSTOPSIG, WIFCONTINUED.

— Built-in Function: **WUNTRACED** ()  
> Return the numerical value of the option argument that may be
> passed to `waitpid` to indicate that it should also return
> if the child process has stopped but is not traced via the
> `ptrace` system call
>
>
>
> **See also:** waitpid, WNOHANG, WCONTINUE.

— Built-in Function: [err, msg] = **fcntl** (fid, request, arg)  
> Change the properties of the open file fid. The following values
> may be passed as request:
>
> `F_DUPFD`: Return a duplicate file descriptor. `F_GETFD`: Return the file descriptor flags for fid. `F_SETFD`: Set the file descriptor flags for fid. `F_GETFL`: Return the file status flags for fid. The following codes may be returned (some of the flags may be undefined on some systems). `O_RDONLY`: Open for reading only. `O_WRONLY`: Open for writing only. `O_RDWR`: Open for reading and writing. `O_APPEND`: Append on each write. `O_CREAT`: Create the file if it does not exist. `O_NONBLOCK`: Nonblocking mode. `O_SYNC`: Wait for writes to complete. `O_ASYNC`: Asynchronous I/O. `F_SETFL`: Set the file status flags for fid to the value specified by arg. The only flags that can be changed are `O_APPEND` and `O_NONBLOCK`.
>
> If successful, err is 0 and msg is an empty string.
> Otherwise, err is nonzero and msg contains a
> system-dependent error message.

— Built-in Function: [err, msg] = **kill** (pid, sig)  
> Send signal sig to process pid.
>
> If pid is positive, then signal sig is sent to pid.
>
> If pid is 0, then signal sig is sent to every process
> in the process group of the current process.
>
> If pid is -1, then signal sig is sent to every process
> except process 1.
>
> If pid is less than -1, then signal sig is sent to every
> process in the process group -pid.
>
> If sig is 0, then no signal is sent, but error checking is still
> performed.
>
> Return 0 if successful, otherwise return -1.

— Built-in Function: **SIG** ()  
> Return a structure containing Unix signal names and their defined values.
